# Understanding the Secret of SARS-CoV-2 Variants of Concern/Interest and Immune Escape

**DOI:** 10.3389/fimmu.2021.744242

**Published:** 2021-11-05

**Authors:** Fuxing Lou, Maochen Li, Zehan Pang, Lin Jiang, Lin Guan, Lili Tian, Jiaming Hu, Junfen Fan, Huahao Fan

**Affiliations:** ^1^ College of Life Science and Technology, Beijing University of Chemical Technology, Beijing, China; ^2^ Tandon School of Engineering, New York University, New York, NY, United States; ^3^ Institute of Cerebrovascular Disease Research and Department of Neurology, Xuanwu Hospital of Capital Medical University, Beijing, China

**Keywords:** SARS-CoV-2 variants, immune escape, vaccine, neutralizing antibody, variants of interest, variants of concern

## Abstract

The global pandemic of the coronavirus disease 2019 (COVID-19), caused by severe acute respiratory syndrome coronavirus 2 (SARS-CoV-2), places a heavy burden on global public health. Four SARS-CoV-2 variants of concern including B.1.1.7, B.1.351, B.1.617.2, and P.1, and two variants of interest including C.37 and B.1.621 have been reported to have potential immune escape, and one or more mutations endow them with worrisome epidemiologic, immunologic, or pathogenic characteristics. This review introduces the latest research progress on SARS-CoV-2 variants of interest and concern, key mutation sites, and their effects on virus infectivity, mortality, and immune escape. Moreover, we compared the effects of various clinical SARS-CoV-2 vaccines and convalescent sera on epidemic variants, and evaluated the neutralizing capability of several antibodies on epidemic variants. In the end, SARS-CoV-2 evolution strategies in different transmission stages, the impact of different vaccination strategies on SARS-CoV-2 immune escape, antibody therapy strategies and COVID-19 epidemic control prospects are discussed. This review will provide a systematic and comprehensive understanding of the secret of SARS-CoV-2 variants of interest/concern and immune escape.

## Introduction

The coronavirus disease 2019 (COVID-19), caused by severe acute respiratory syndrome coronavirus 2 (SARS-CoV-2), broke out in Wuhan, China in December 2019. Since then this epidemic has been spread and intensified internationally and defined as a Public Health Emergency of International Concern (PHEIC) by the World Health Organization (WHO) on January 30, 2020. As of September 26, 2021, 231,614,338 cases of COVID-19 have been confirmed, including 4,744,918 deaths caused by SARS-CoV-2-induced inflammatory infections or other complications [https://coronavirus.jhu.edu/map.html].

**Figure 1:**
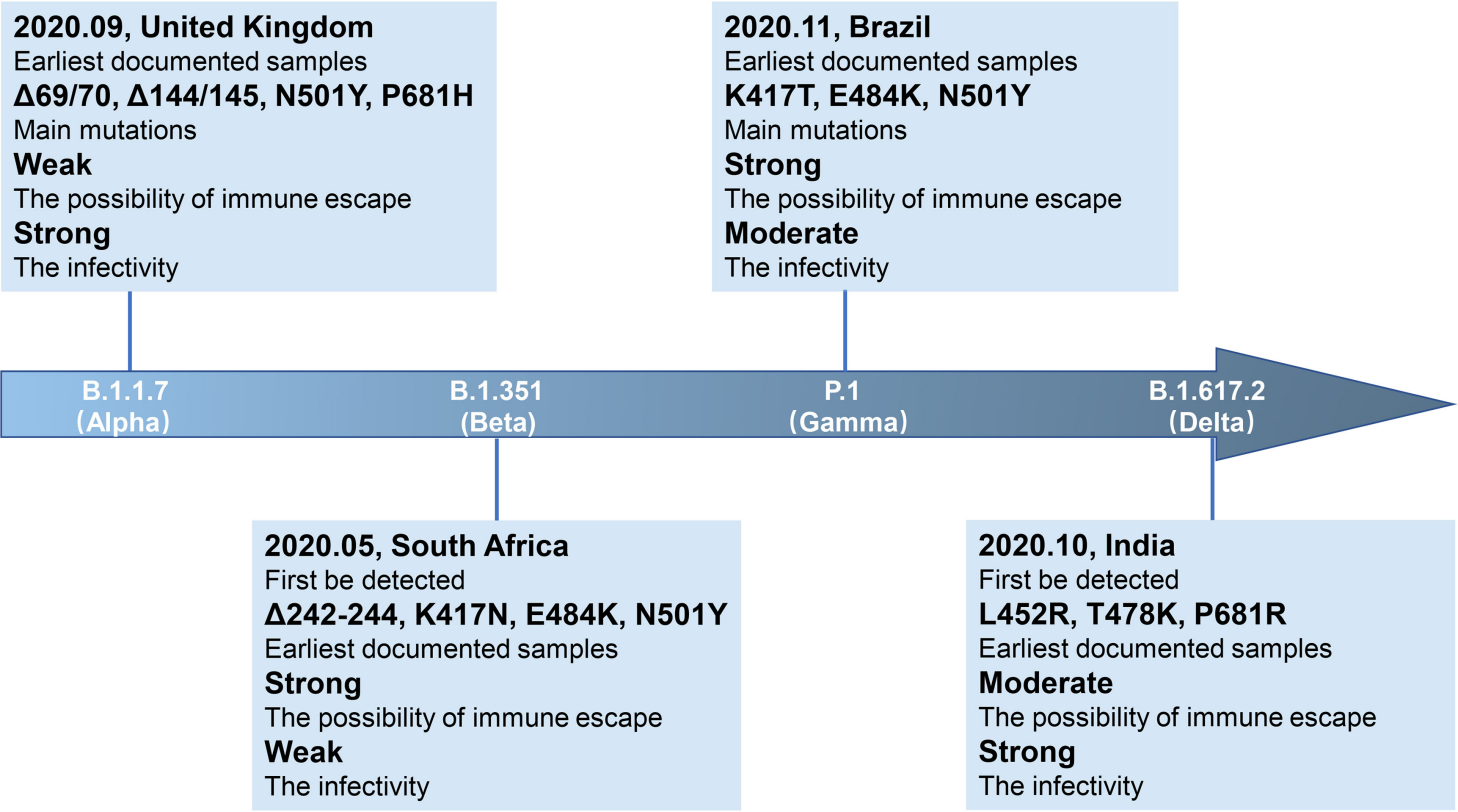
Brief information of four VOC variants. Four VOC variants (B.1.1.7, B.1.351, P.1, and B.1.617.2) are marked in the arrow according to the date of designation, and their related brief information (e.g., the time and location of earliest documented samples, infectivity, main mutations, immune escape ability) are displayed in the corresponding location.

**Figure 2:**
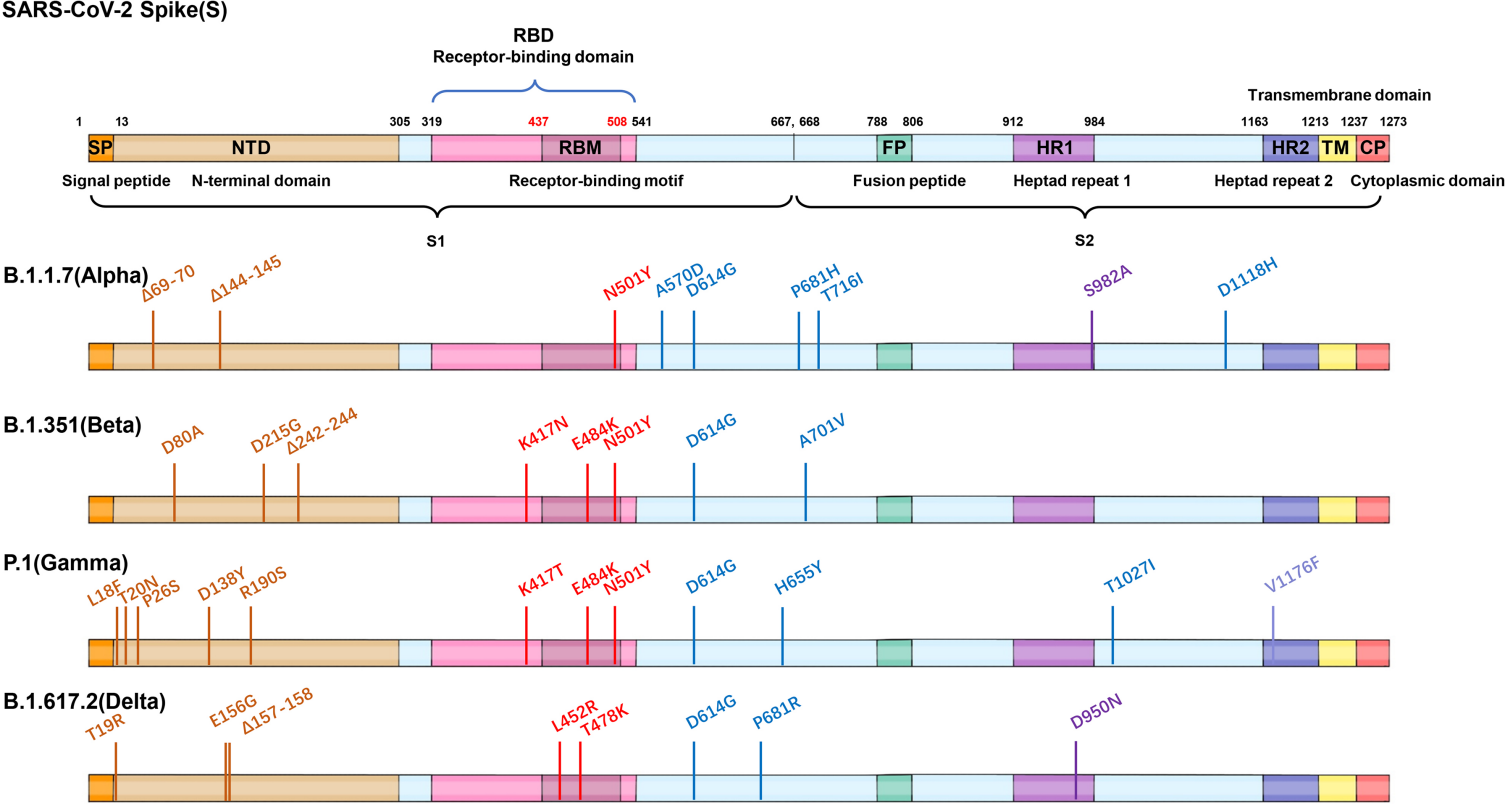
Mutations of four VOC variants. Schematic showing the locations of amino acid substitutions of four VOCs (B.1.1.7, B.1.351, P.1, and B.1.617.2) in spike protein. The RBD region is shown in modena, the NTD region is shown in shallow orange.

**Figure 3:**
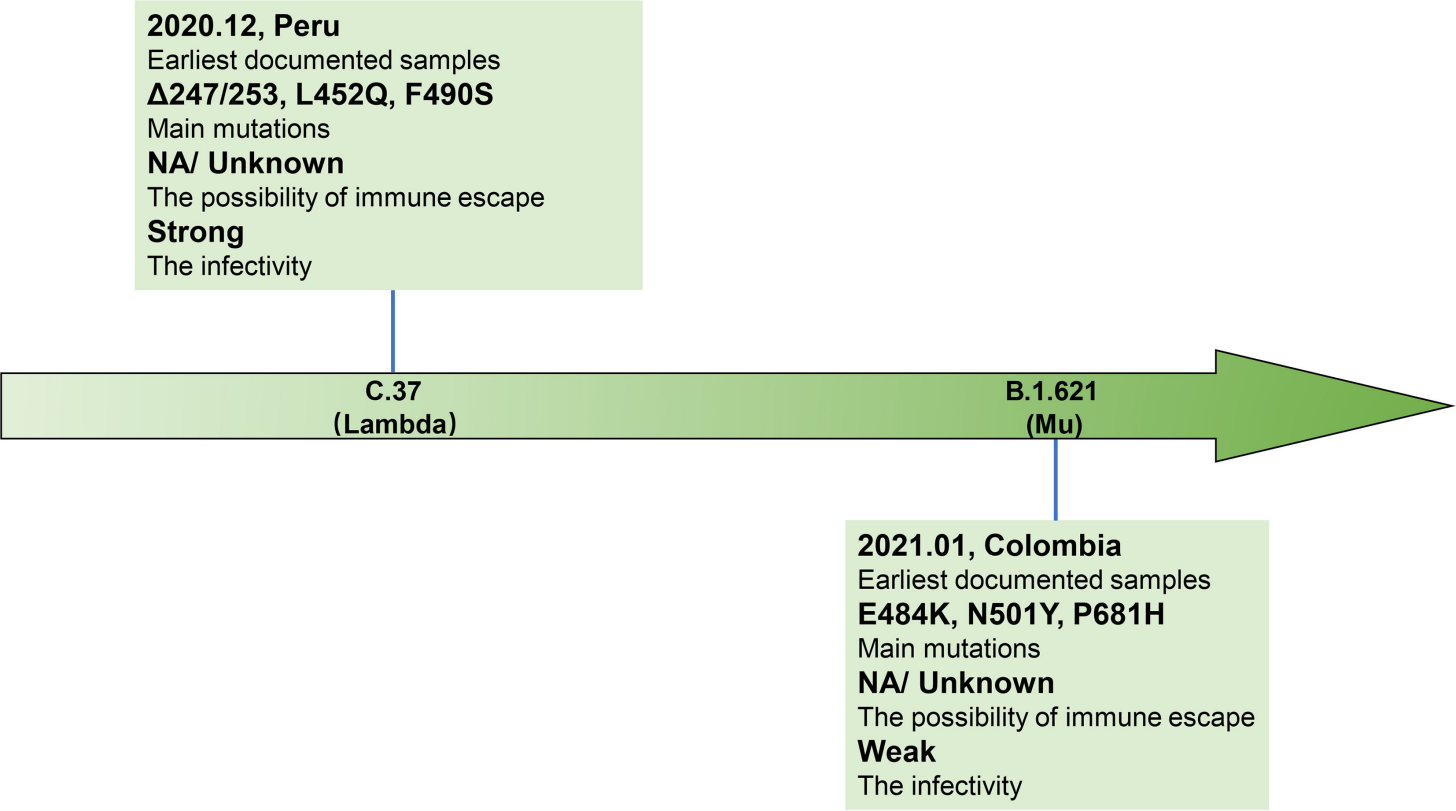
Brief information of two VOI variants. Two VOI variants (C.37 and B.1.621) are marked in the arrow according to the date of designation, and their related brief information (e.g., the time and location of earliest documented samples, infectivity, main mutations, immune escape ability) are displayed in the corresponding location.

**Figure 4:**
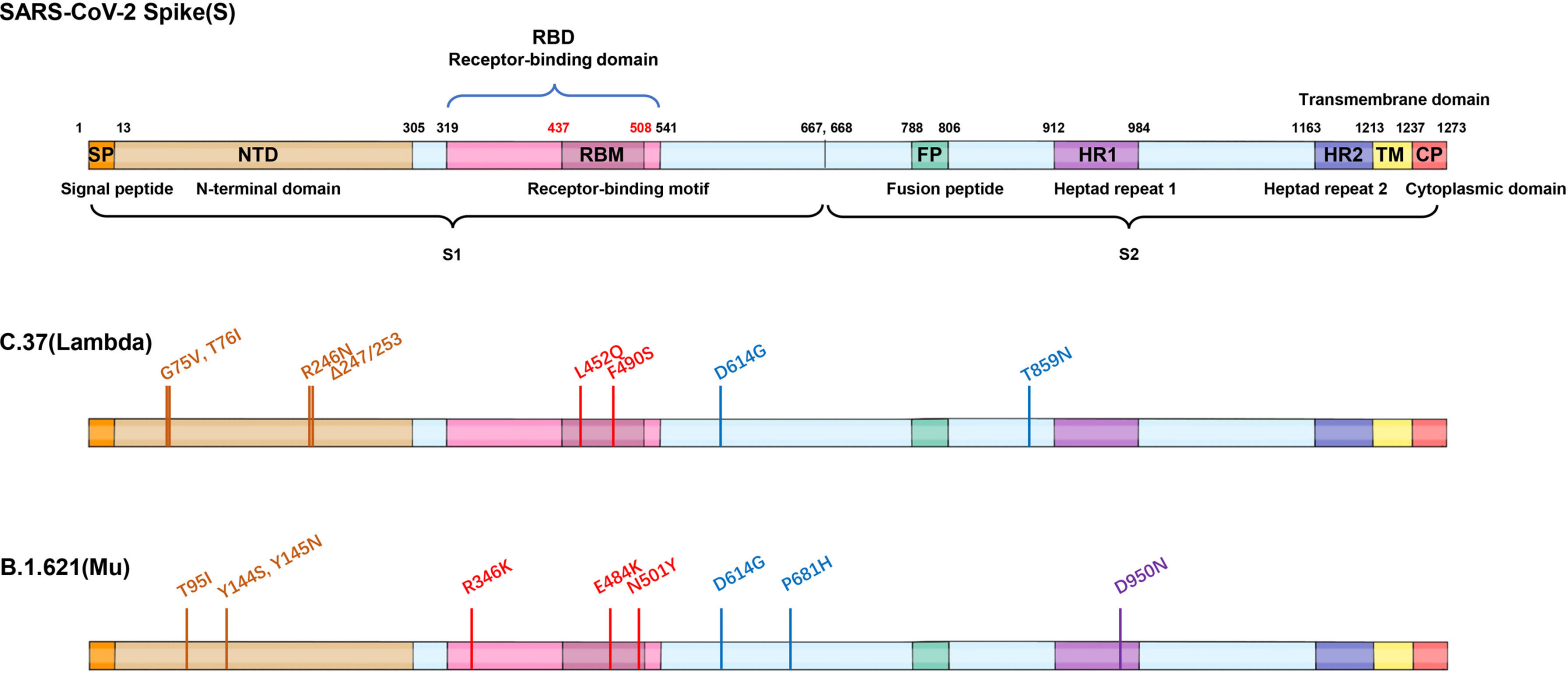
Mutations of two VOI variants. Schematic showing the locations of amino acid substitutions of two VOI variants (C.37 and B.1.621) in spike protein. The RBD region is shown in modena, the NTD region is shown in shallow orange.

SARS-CoV-2 is a single-stranded RNA virus, typical symptoms of infected patients are fever, cough, chest discomfort, and respiratory distress syndrome (RDS) often occurs in severe cases (1). The inherent error-prone characteristics of viral RNA-dependent RNA polymerase (RdRp) result in the random introduction of mutations in the viral genome during replication. Although the virus encodes an exonuclease (ExoN, nsp14) with a proofreading function, it cannot eliminate the occurrence of viral mutations (2, 3). With continued spreading and viral replication, chronic infection will increase the possibility of virus adaptive mutation (4, 5). The multiple emerging mutations of SARS-CoV-2 variants confer worrisome epidemiologic, immunologic, or pathogenic characteristics (6). WHO developed a Variant Classification scheme, and the categories of variants of concern (VOC) and variants of interest (VOI) were proposed. VOC refers to the SARS-CoV-2 variants that can increase the transmissibility or cause detrimental change in COVID-19 epidemiology by strongly impairing the effectiveness of vaccines and neutralizing antibodies (**Table 1**), or can cause more serious disease conditions. VOI means the SARS-CoV-2 variants that harbor mutations which are predictable or known to affect viral characteristics, such as infectivity, disease severity, immune escape, or showing a sudden risk to global public health security [https://www.who.int/en/activities/tracking-SARS-CoV-2-variants/]. At present, there are mainly four kinds of VOC: B.1.1.7 (Alpha, originated in the United Kingdom), B.1.351 (Beta, originated in South Africa), P.1 (Gamma, originated in Brazil), and B.1.617.2 (Delta, originated in India) (**Figures 1**, **2**). VOI mainly includes C.37 (Lambda, first detected in Peru) and B.1.621 (Mu, first detected in Colombia) (**Figures 3**, **4**) [https://www.who.int/en/activities/tracking-SARS-CoV-2-variants/].

**Table 1:** The transmissibility and immune escape ability of four VOCs.

VOC	Transmissibility	Immune escape ability
B.1.1.7	Harbored 43–90% higher reproduction number than WT (7).	The impact of EUA monoclonal antibodies on B.1.1.7 is negligible [https://www.regeneron.com/downloads/treatment-covid19-eua-fact-sheet-for-hcp.pdf] [https://www.fda.gov/media/145802/download] and the effect of the neutralization of convalescence serum and vaccine-immune serum on B.1.1.7 is slight (8, 9).
B.1.351	B.1.351 is 50% more transmissible than previously circulating variants in South Africa (10).	The neutralization activity of bamlanivimab and etesevimab together against B.1.351 decreased by 215-fold [https://www.fda.gov/media/145802/download], and the neutralization activity for casirivimab and imdevimab was remained [https://www.regeneron.com/downloads/treatment-covid19-eua-fact-sheet-for-hcp.pdf]. The neutralization effects of convalescence serum and vaccinated serum against B.1.351 decreased sharply (11, 12).
B.1.617.2	The increase in the effective reproduction number compared with the Alpha variant (B.1.1.7) is estimated to be 55% (13).	Among the four EUA monoclonal antibodies, bamlanivimab lost its neutralization activity against B.1.617.2 (14). The neutralization ability of convalescent serum and vaccine serum is weakened (15)
P.1	1.7- to 2.4-fold more transmissible than WT strain (16).	The neutralization activity of bamlanivimab and etesevimab together against P.1 decreased by >46-fold, and the neutralization of casirivimab and imdevimab together was remained [https://www.fda.gov/media/145802/download] [https://www.regeneron.com/downloads/treatment-covid19-eua-fact-sheet-for-hcp.pdf]. Reduced neutralization of convalescent and vaccine-immune serum (17).

Variants Under Monitoring (VUM) means a SARS-CoV-2 variant with specific mutations that affect virus characteristics, and may pose a potential threat in the future, but there is no definitive evidence of phenotypic or epidemiological impact at present [https://www.who.int/en/activities/tracking-SARS-CoV-2-variants/]. Currently designated VUMs include B.1.427/B.1.429 (first detected in California, USA), R.1 (first detected in several countries in January 2021), B.1.466.2 (first detected in Indonesia), B.1.1.318 (detected in multiple countries in January 2021), B.1.1.519 (detected in several countries in January 2021), C.36.3 (detected in several countries in January 2021), B.1.214.2 (detected in several countries in November 2020), B.1.1.523 (detected in several countries in May 2020), B.1.619 (detected in several countries in May 2020), B.1.620 (detected in several countries in November 2020), C.1.2 (first detected in South Africa), B.1.525 (Eta, detected in several countries in December 2020), B.1.526 (Iota, first detected in the United States), and B.1.617.1 (Kappa, first detected in India) [https://www.who.int/en/activities/tracking-SARS-CoV-2-variants/]. Some former VOIs but no longer designated as VUMs include P.2 (first detected in Brazil) and P.3 (first detected in the Philippines) (**Supplementary Figures 1**, **2**) [Variants: distribution of cases data, 20 May 2021-GOV.UK (www.gov.uk)].

In this article, we provide a systematic and comprehensive summary of the key genetic variants of SARS-CoV-2 and elucidate the impacts of pivotal mutations on viral transmissibility, infectivity, and immune escape of vaccines and antibodies.

## Major Genetic Variants of SARS-CoV-2

B.1.1.7 is the earliest prevalent variant, which was originally identified in the United Kingdom in September 2020 (18), and has a significant transmission superiority with a higher reproduction number (R) than non-VOC lineages (19). On December 18, 2020, it was designated by the Public Health England (PHE) as a VOC lineage [https://researchportal.phe.gov.uk/en/]. This variant with 10 mutations in spike (S) protein (20, 21) can be divided into three subgroups. The major strain contains Δ69-70, and the other two subgroups lack this deletion, which indicated that Δ69-70 obtained selective advantages in the variation process of SARS-CoV-2 (22). As of January 13, 2021, there were 76 confirmed cases of B.1.1.7 in 12 states in the United States (23), and this variant has been circulating in 174 countries so far, indicating that B.1.1.7 is highly contagious [https://cov-lineages.org/]. According to the survey of PHE, B.1.1.7 would lead to a 30%-50% increase in secondary attack rate (18). In some studies, convalescent plasma and vaccine sera were applied in B.1.1.7 neutralization assays, and no widespread immune escape was observed (22). Pseudovirus neutralization assay showed that the neutralization of BNT162b2-immune sera against B.1.1.7 was largely preserved, indicating that the variants have difficulty escaping from vaccine-mediated immune protection (20). Wang et al. evaluated the neutralization effect of two vaccines BBIBP-CorV (Sinopharm) and CoronaVac (Sinovac) developed in China against B.1.1.7. The results showed that compared with wildtype (WT), the BBIBP-CorV-immune serum remained neutralizing potency to B.1.1.7, but the geometric mean titers (GMTs) of CoronaVac-immune serum against B.1.1.7 decreased significantly (24). The above studies suggested that B.1.1.7 did not pose a great threat to the protective efficacy of COVID-19 vaccines. However, B.1.1.7 variant infection was related to higher virus titer in nasopharyngeal swabs, which accounted for the increased mortality (25). Therefore, the necessity of continuous SARS-CoV-2 sequence surveillance should be highlighted.

**Figure 5:**
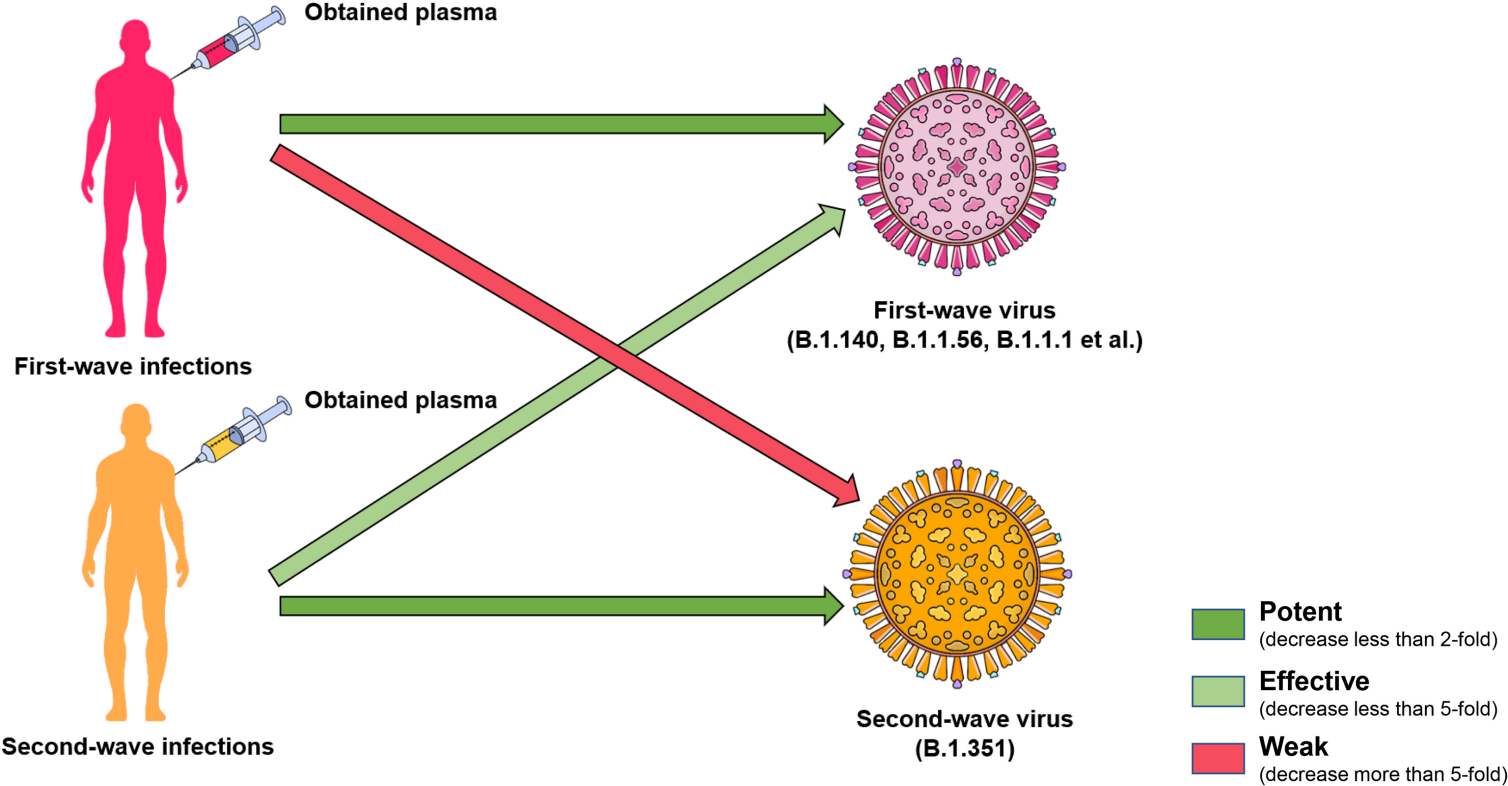
Cross neutralization of convalescent plasma. The cross neutralization of convalescent serum has been noticed by the neutralization activity test. The convalescent serum from B.1.351-infected patients retained the neutralization against the virus in the first-wave epidemic in South Africa. However, the serum obtained from the first-wave of epidemic infection could not effectively neutralize B.1.351. The green arrow indicates that the neutralization still remains, and the light green means a worse neutralization effect. Pink indicates a significant decrease in neutralization efficacy.

B.1.351 was discovered in South Africa in May 2020, and then expanded rapidly to become the dominant lineage in South Africa. It was related to the sharp increase of infected cases nationwide in mid-December, which strongly indicated that this variant had a selective advantage (26). Through the analysis of virus sequencing, it was found that B.1.351 also has three subgroups: 501Y.V2-1, 501Y.V2-2, and 501Y.V2-3. Compared with the receptor binding domain (RBD) and N terminal domain (NTD) sequences of the other two subgroups, 501Y.V2-3 contains R246I mutation and lacks L18F mutation, implying the evolution of SARS-CoV-2. The RBD of B.1.351 harbors three notable mutation sites: K417N, E484K and N501Y, and the combined effect of these three mutations could enhance the affinity of viral spike protein to ACE2 (27). A previous study showed that 21 of 44 convalescent plasma samples lost neutralizing activity against B.1.351 (28). Pseudovirus neutralization assay confirmed that 12 of 17 monoclonal antibodies were ineffective against B.1.351 (29). Compared with WT, the 50% plaque reduction neutralization titer (PRNT_50_) of 14 convalescent serum against live B.1.351 virus decreased by 3.2- to 41.9-fold (30). The impaired efficacy of vaccine-immune sera including mRNA-1273 (Modena) and BNT162b2 (Pfizer) against 501Y.V2 was demonstrated (11), and 20 of 25 BBIBP-CorV (Sinopharm) vaccinated serum samples showed complete or partial neutralization loss against B.1.351, and the CoronaVac vaccinated sera showed a significant decrease of GMTs against B.1.351, accompanied by the complete or partial neutralization loss of most samples (24). The efficacy of the ChAdOx1 nCoV-19 vaccine (AZD1222) against symptomatic infection caused by B.1.351 was only 10.4%, which directly led to the suspension of the ChAdOx1 nCoV-19 vaccine in South Africa (12). The neutralizing activity against B.1.351 of three potent monoclonal antibodies (2-15, LY-CoV555, and REGN10933) approved for emergency use also decreased significantly (11). These studies raised concerns about the efficacy of vaccines and antibodies against B.1.351. Interestingly, a study carried out in South Africa showed that the convalescent serum from B.1.351-infected patients retained the neutralization activity against the virus in the first-wave epidemic with only a 2.3-fold decrease (30). However, the serum obtained from the first wave of the epidemic infection could not effectively neutralize B.1.351 (30). These findings suggested that the sera from individuals infected with B.1.351 possess cross-neutralization activity to other variants, and the antibodies elicited by the variants with stronger immune escape ability may have more extensive neutralization ability (**Figure 5**). On the other hand, in the presence of cross-neutralization activity, the neutralization efficacy of antibodies induced by original strains against other strains still decreased to some extent. Therefore, the existence of the cross-neutralization provided an idea for vaccine optimization. Universal vaccines using variants in the epidemic as seed strains against multiple variants could quickly and effectively restrain the spread of the epidemic (21).

In November 2020, a second wave of COVID-19 epidemic broke out in Brazil, causing 76% of the population to be infected (31), which was similar to the epidemic in South Africa. After virus sequence analysis, the variant was named P.1. The RBDs of P.1 and B.1.351 contain mutations in site 417, 484, and 501 residues, except that P.1 harbors K417T, B.1.351 harbors K417N. In addition, the L18F mutation located in the NTD was shown to be related to immune escape potentiality (32). Because of its strong infectivity, P.1 was designated as VOC by WHO on January 11, 2021 (20, 33). The transmissibility of P.1 could be 1.7-fold to 2.4-fold higher than that of non-P.1 lineages, and the convalescent serum from non-P.1 SARS-CoV-2 infected patients could only provide 54%-79% protection against the infection of P.1 (16). According to data from WHO, a variant named P.2 with E484K mutation was originally sequenced in Brazil in April 2020 [https://www.who.int]. P.2 variant showed significant resistance to the vaccinated serum, as the neutralization efficacy of BNT162b2 (Pfizer) against the Brazilian/Japanese P.2 strain decreased 5.8-fold and mRNA-1273 (Moderna) decreased 2.9-fold. Similarly, the vaccine neutralizing activity against the Brazilian/Japanese P.1 strain also decreased significantly (6.7-fold for BNT162b2 and 4.5-fold for mRNA-1273) (17). Few studies have been published on P.2 since it did not cause large-scale outbreaks in Brazil and other countries, but further analysis of the sequence of P.1 and P.2 may reveal the evolution of the virus. Another SARS-CoV-2 variant named P.3 was reported in the Philippines in March 2021. This lineage has several notable mutations in the S protein, including E484K, N501Y, and P681H (**Supplementary Figures 1**, **2**). P.3 as well as P.1 belong to B.1.1.28 lineage (https://www.who.int/en/activities/tracking-SARS-CoV-2-variants/).

With increased infectivity and virulence, a new variant with an L452R mutation in S protein appeared in California in March 2020, which was named B.1.427/429. Since October 2020, a peak of the COVID-19 epidemic occurred in southern California. According to the sequence analysis of local strains, it was found that most of the variants came from clade 20C (34), which first emerged in Europe and then mutated in Britain and other places, becoming the most widely distributed variant B.1.1.7. From September 2020 to January 2021, 2172 nasal/nasopharyngeal swabs from 44 counties in California were sequenced and it was found that the B.1.427/429 positive sample ratio increased from 0% to more than 50% (34). There are four missense mutations in the S protein of B.1.427/B.1.429, including L452R, S13I, W152C, and D614G, among which the L452R mutation is located in the RBD region (**Supplementary Figures 1**, **2**). It was found that the virus shedding amount of this variant *in vivo* was 2-fold higher than that of WT, and the production of pseudovirus containing L452R in cell culture and lung tissue also increased, but was lower than that of B.1.1.7, B.1.351, and P.1, which showed that this variant had higher infectious ability (35). Furthermore, B.1.427/B.1.429 had a certain immune escape ability, which was demonstrated by the significantly reduced neutralization ability of plasma from Pfizer/BioNTech BNT162b2 or Moderna mRNA-1273 vaccinated participants and impaired effects of convalescent serum from patients against B.1.427/B.1.429 (36). Worryingly, more mutations may be accumulated on the basis of B.1.427/B.1.429 in the future, which would further increase the possibility of immune escape (37).

A previous VOI, currently designated VUM variant B.1.526 was first identified in the New York region in November 2020, and began to spread at an alarming rate (38). The most significant mutations in the spike of this lineage are L5F, T95I, D253G or S477N, and D614G (39) (**Supplementary Figure 2**). Preliminary data analyzed by the New York City Department of Health and Mental Hygiene (DOHMH) suggested that the B.1.526 variant was not associated with an increased risk of breakthrough infection or reinfection after vaccination and with more serious disease conditions (38). The antibodies elicited by infection and vaccines (Pfizer BNT162b2 and Moderna mRNA-1273) could retain the complete neutralization titer against B.1.526 harboring S477N, but the neutralization was 3.5-fold lower against B.1.526 harboring E484K than that of D614G strain (40). In addition, the titer of E484K neutralized by REGN10933 monoclonal antibody decreased by 12-fold, but the neutralization activity of its combined cocktail with REGN10987 against B.1.526 was completely retained (40). Other studies also evaluated the resistance of B.1.526 variant to neutralizing antibodies and ACE2 blocking antibodies induced by the mRNA-1273 vaccine within 7 months. These results suggest that current vaccines still retained neutralization ability for B.1.526 and the variant did not show widespread immune escape (41).

B.1.525, also known as 20A/S: 484K, was discovered and expanded rapidly in many countries in December 2020 (https://covariants.org/variants/20A.S.484K). Phylogenetic analysis showed that the lineage originated from Nigeria (42). Although it did not circulate all over the world, it was defined as the lineage of international significance (43) and was classified as VUM for the possibility of increasing infectivity, virulence and reducing the effectiveness of the vaccine by notable mutations carried by the variant (https://www.who.int/en/activities/tracking-SARS-CoV-2-variants/). Eight mutations (D614G, Q677H, E484K, F888L, A67V, Δ69/70, Δ144/145, and Q52R) in the Spike protein of B.1.525 were identified (**Supplementary Figure 2**). Q677H harbored by B.1.525 can regulate the transmissibility (42). E484K exists in B.1.351, P.1, and P.2 variants and is related to immune escape. Δ69/70 and Δ144/145 were detected in B.1.1.7, Δ69/70 was shown to have a selective advantage (42), and 144/145 site was verified to be a binding epitope of multiple antibodies targeting NTD (36). The average viral load of the upper respiratory tract between this variant and B.1.1.7 infected patients was similar (43). At the same time, B.1.525 was resistant to neutralization of convalescent serum, vaccine-elicited serum, and monoclonal antibodies (44).

As of September 26, 2021, India’s confirmed cases in has reached 33,652,745, becoming the country with the second largest cumulative number of confirmed cases in the world, which was propelled by variant B.1.617 [https://coronavirus.jhu.edu/map.html]. Through genome sequencing of local confirmed cases, it was found that the local epidemic variant B.1.617 harbored E484K, L452R, and P681R mutations, all of which have been present in other epidemic strains. E484K was detected in B.1.351 while the 681 residue was detected as H681 in B.1.1.7. This showed that the B.1.617 may obtain the characteristics of both B.1.1.7 and B.1.351, and it was considered as one of the most concerned epidemic variants (45). With the spread of the local epidemic in India, B.1.617.1, B.1.617.2, and B.1.617.3 strains emerged successively. B.1.617.2, named Delta, was first sequenced in India in October 2020 and then spread globally. It posed a greater threat to global public health security than the other two lineages and was recognized as VOC by WHO on May 11, 2021 [https://www.who.int/publications/]. At present, at least 163 countries were under the shadow of B.1.617.2 [https://outbreak.info/situation-reports]. Under the background of the prevalence of B.1.1.7, B.1.617.2 strain also appeared in the United Kingdom, and gradually increased or even became a dominant strain, indicating that this variant had a significant competitive advantage (46). In May 2021, a local outbreak caused by Delta occurred in Guangzhou, China, where the variant spread four generations in only 10 days, indicating its remarkable infectivity. A research team collected the clinical information of 159 Delta infection cases in Guangzhou, China and analyzed their clinical characteristics and viral dynamics. Compared with the WT strain, the Delta strain harbored a significantly shorter median incubation period (4 days vs. 6 days), and was featured with a higher viral load (median Ct 20.6 vs. 34.0). Moreover, patients with Delta infection were associated with a shorter time of the deterioration to critical illness, a higher risk of critical status and longer period for RNA-negative conversion than WT (47). These clinical data confirmed that Delta carried unique properties that require continuous monitoring and follow-up.

B.1.617.2 with Δ156-157, G158R, L452R, T478K and other mutations is considered to pose great challenges to the effectiveness of vaccines and neutralizing antibodies (48). Compared with the WT, the neutralizing antibody titers (NAbTs) of sera from BNT162b2 recipients against B.1.617.2 reduced by 5.8-fold (49), and the neutralization titer of serum from Pfizer Comirnaty vaccine had a 3-fold decrease against B.1.617.2 compared with B.1.1.7 (14). Serum from participants injected with a single dose of AstraZeneca vaccine almost completely lost neutralizing activity against B.1.617.2. The effective dose 50% (ED50) analysis showed that compared with B.1.1.7, the neutralization titer of patients sera in 6 and 12 months after infection against B.1.617.2 decreased by 4- to 6-fold, respectively (14). In addition, a recent study held by PHE showed that even after two doses of vaccine, recipients could still be infected by B.1.617.2. Among the four clinically approved antibodies Bamlanivimab, Etesevimab, Casirivimab, and Imdevimab, Bamlanivimab lost its neutralization activity against B.1.617.2, which is considered to be caused by the mutation at site L452, while the other three antibodies remained neutralizing activity (14). Therefore, it is still necessary to monitor the efficacy of the vaccines and antibodies against circulating variants.

Recently, a new SARS-CoV-2 variant C.37 had infected more than 80% of the population in Peru (14). C.37 has a similar mutation as B.1.617.2 in 452 site (L452Q) as well as a mutation F490S in the antibody-binding epitopes of RBD, which may reduce the neutralization of partial RBD antibodies. WHO named the variant as Lambda and designated it as a variant of interest in June 2021 [https://www.who.int/en/activities/tracking-SARS-CoV-2-variants/]. Given C.37 may have higher contagious and immune escape ability, it is necessary to take continuous surveillance for the Lambda variant.

The SARS-CoV-2 Mu variant (B.1.621), a new variant of interest classified by WHO on August 30, 2021, has been detected in at least 50 countries, predominantly in Colombia [https://outbreak.info/situation-reports]. In the context of Gamma’s dominance, Mu transcended Gamma within only five months, propelling the epidemic in Colombia, implying the conspicuous infectivity. The Mu variant was first detected on August 1, 2021 with nine mutations (D614G, P681H, R346K, N501Y, E484K, T95I, D950N, Y145N, Y144S) in the Spike protein (**Figure 4**). The distinctive substitutions distributed in NTD (T95I, Y145N, Y144S), RBD (R346K, N501Y, E484K) and furin cleavage site (P681H) were associated with increased viral infectivity and immune escape ability. Pseudovirus neutralization assay showed that the neutralization titers of BNT162b2-vaccinated sera against Mu strain were reduced by 7.6-fold compared with WT, showing significantly more resistant than other VOCs (2.6-fold against Alpha; 8.2-fold against Beta; 4.1-fold against Gamma; 4.0-fold against Delta) and VOIs (3.4-fold against Lambda), and a similar situation also existed in convalescent sera (50).

SARS-CoV-2 variants appeared frequently all over the world, which aroused great global attention. According to previous studies, climatic factors will change the speed of virus transmission to a certain extent and affect the spread of the epidemic (51). However, with highly contagious characteristics of SARS-CoV-2, it seems that the regulatory role of climate in virus spread is attenuated. If effective control measures are not taken in time, large-scale epidemic may still break out in hot and humid climates (52), which is proved by the emergence of B.1.351 and B.1.617 strains.

## Impacts of Mutations on Transmissibility, Infectivity, and Immune Escape

Spike protein of SARS-CoV-2 contains N-terminal domain (NTD), receptor binding domain (RBD), and other regions (such as fusion peptide region) (1). The RBD and NTD regions are mainly located on the S1 subunit, while the fusion peptides and other regions are located on the S2 subunit. Based on the sequence analysis of the main epidemic strains (B.1.1.7, B.1.351, P.1, B.1.427/429, and B.1.617), it was found that the main mutations located in the RBD region are K417N/T, N439K, L452R, E484K/Q, and N501Y. The mutations located in the NTD region are mainly as follows: L18F, T20N, P26S, Δ69-70, D80A, D111D, D138Y, G142D, Δ144, W152G, R190S, D215G, and Δ242-244; other regions are A570D, D614G, H655Y, P681H/R, A701V, T716I, S982A, and T1027I (22, 26, 29, 45, 53, 54) (**Figures 2**, **4**).

### Mutations in the RBD Region

The N439K mutation located in the receptor binding motif (RBM) was first found in Scotland in March 2020 and spread widely in European countries (4). N439K is thought to enhance the binding of RBD to ACE2 (55) and escape the neutralization of some monoclonal and polyclonal antibodies in the convalescent serum (4). Another amino acid mutation in the RBM region, Y453F, can also enhance the affinity of the virus to ACE2 (55). It is worth noting that the virus in all patients infected with SARS-CoV-2 is associated with minks harboring Y453F strain (56).

The serum analysis of nearly 650 recovered patients with SARS-CoV-2 infection showed that 90% of the neutralizing antibodies targeted the RBD region (57), which may be due to the lack of glycan shielding in the amino acids in the RBD region compared with other regions (58). K417N/T was detected in B.1.351 variant and P.1 variant, and 417 residue was a potential key site for immune escape (4, 28). VH3-53/66 encodes a class of common and effective neutralizing antibodies, and K417N mutation could reduce the affinity of these antibodies to S protein (28, 58, 59). Different from N501Y and E484K, which belong to the same RBD region, no evidence for positive selection of K417N was demonstrated (26). K417N mutation both in B.1.351 and P.1 damaged the affinity between them (4, 55, 60), whereas the E484K and N501Y mutations increased the interaction between S protein and ACE2 (5). Furthermore, the effect of virus mutation on neutralizing antibodies may have a cumulative effect, the more mutation sites, the lower neutralizing response of antibodies (26). Thus, timely and effective prevention and control measures should be taken to restrain the spread of the epidemic and thus reduce the emergence of new variants.

L452R was first found in a novel lineage in California named CAL.20C (34), and then detected in B.1.617 [https://www.gisaid.org/], which made the conformation of the S protein more stable (54), leading to the increased affinity of the virus and ACE2 (2). L452 residue did not directly interact with ACE2, but the L452R mutation could affect the structural stability of the region where S protein interacts with ACE2 and facilitate SARS-CoV-2 to enter into human respiratory organs (37), which accounted for the prevalence of B.1.427/429 in North America and B.1.617 in India. In addition, the mutation in this site seemed to have a positive effect on immune escape, as the mutation of L452 residue may induce the conformational change of RBD, thus reducing the binding ability of several monoclonal neutralizing antibodies (2, 61–63) and diminishing the neutralization activity of convalescent sera (37, 61). Single L452R mutation could reduce or abolish the neutralizing activity of clinical-stage monoclonal antibodies such as regdanvimab (CT-P59), etesevimab (LY-CoV016), and bamlanivimab (LY-CoV555) (36). L452R has also been confirmed to reduce the neutralizing activity of some antibodies, which do not directly bind to the ACE2-binding epitopes, and it is considered to be a moderate immune escape site for these antibodies (64). The presence of L452R mutation in multiple lineages and regions indicated that this mutation has a positive selection, which may be due to the selective pressure of RBD-specific neutralizing antibodies (36).

All three variants B.1.1.7/B.1.351/P.1 contain N501Y mutation [https://cov-lineages.org/], and this mutation could enhance the affinity of virus S protein with ACE2, especially with the side chains of residues Y41 and K353 of ACE2 (18, 20, 22, 55, 65–67). In addition, the N501Y mutation enabled the virus to infect BALB/c mice, which expanded its host range (67). The neutralization ability of the serum inoculated with Pfizer BNT162b2 vaccine against pseudovirus with N501Y was almost the same as that of the pseudovirus without the mutation (20, 68). However, in the presence of E484K, N501Y, and K417N, the neutralization activity of sera from Moderna mRNA-1273 or Pfizer BNT162b2 vaccinated individuals decreased to a certain extent (63). These findings indicated that a single N501Y mutation is not essential for immune escape, but the accumulation of such key sites will eventually promote immune escape.

The mutation of 484 site exists in the form of K484 in B.1.351 and P.1 while Q484 in B.1.617 (https://cov-lineages.org/) (45). 484 site may also be one of the important immune dominant epitopes. As one of the most important amino acid sites in S protein, E484 mutates to K, Q, or P, the antibody neutralization titer decreases more than 10-fold (69). E484K has been shown to reduce the neutralization of convalescent serum and some antibodies (69). In addition, further mutation of E484K on B.1.1.7 will further reduce the serum neutralizing response of BNT162b2 vaccines (8). Some antibodies from IGHV3-53 and IGHV3-66 genes target the E484 residue of SRAS-CoV-2 S protein, and the mutation at position 484 has a negative effect on the neutralization of these antibodies (28, 70). The 484 site mutation also reduced the neutralizing activity of a variety of monoclonal antibodies in clinical stage, including REGN10933 and LY-CoV-555 (2). Some studies have confirmed that the mutation of E484K could eliminate the key interaction between epitope antibodies against 484 and Arg50 or Arg96, resulting in decreased antibody neutralization efficiency (71, 72). In contrast, Brii-196, COV2-2130, P2C-1F11, and H014 still preserved high neutralization ability, which may be due to their broad-spectrum antigen-binding epitopes (33, 53, 73). The above results provided a novel approach for the future antibody cocktail therapy, which means that the antibody cocktail against different immune epitopes could improve the overall neutralization activity. Although the Indian variant did not contain mutations at sites 417 and 501, given that India currently has the second-largest number of infected people in the world, the E484K mutation may occur on the main epidemic strain B.1.617.2 in India. And it will further improve the affinity to ACE2 and immune escape ability of B.1.617.2, thus improving its infectivity. Antibody neutralizing response was mainly affected by a few dominant epitope mutations in RBD. E484 position, the targeting site of antibodies such as heavy chain germline IGHV3-53 and IGHV3-66, has the greatest influence on antibody binding and neutralization in RBD region (54, 62, 69, 74–76). E484K can escape not only the neutralization of monoclonal antibodies C121, C144 (77) and the serum from convalescent patients (72), but also the neutralization of the combination of REGN10989 and REGN10934 monoclonal antibody cocktail (78).

### Mutation of NTD Region

The deletion of fragments in the NTD region of SARS-CoV-2 was repeatedly observed in the process of evolution, and included sites being considered to be related to immune escape such as L18F and R246I mutations (32, 33, 72).

B.1.1.7 harbored two mutations in the NTD region, Δ69-70 and Δ144. As mentioned earlier, strains with Δ69-70 occupied the dominant position in the middle and later stages of the epidemic in the UK, indicating that these mutations are positive selective. Δ69-70 was speculated to change the conformation of the exposed NTD loop and increase the infectivity of the virus (79). Δ144 significantly reduced the neutralization of most antibodies targeting NTD against B.1.1.7 variants, indicating that the 144 site was one of the neutralizing epitopes for antibodies targeting NTD (33).

B.1.351 and P.1 contain multiple mutations in the NTD region (B.1.351: D80A, D215G, Δ242-244, P.1: L18F, T20N, P26S, D138Y, R190S) [https://cov-lineages.org/]. However, the main targeting site of NTD for antibody against B.1.351 was 242-244 residues, whose deletion reduced the neutralization ability of many kinds of potent antibodies targeting NTD, including 4A8 monoclonal antibody, by more than 1000-fold (33).

The NTD of B.1.427/B.1.429 contains S13I and W152C mutations. This variant achieves neutralization escape through an indirect strategy (36). The S13I mutation could extinguish the integrity of the NTD vulnerable sites by destroying the C15/C136 disulfide bond (36).

The neutralizing potency of the antibody targeting NTD region was poor, greatly reducing the antibody types available for clinical use. However, the mechanisms that determine the antibody failure remain unclear. Present studies have suggested that antibodies targeting NTD may play the role by: (1) blocking the fusion of virus and cell membrane; (2) promoting antibody-mediated cytotoxicity (ADCC); (3) interfering with other coreceptors, such as DC-SIGN and L-SIGN (8, 32, 80). Further studies found that there was a repetitive deletion region (RDR) in the NTD region, which contains most of the immune epitopes of NTD, and the mutation in the RDR region could lead to a decrease in the neutralization ability of antibodies targeting NTD (36). However, Δ144 and Δ242-244 are located in the RDR2 and RDR4 region, respectively, which eliminate the binding of 4A8 targeting RDR2 and RDR4 (33). The existence of the RDR region could explain how antibodies targeting NTD cannot effectively neutralize several mainstream variants. The above analysis showed that multiple immune escape mutations exist in NTD, thereby this region is also in a stage of immune pressure similar to RBD. When studying the antigen drift of new variants, neutralizing antibodies targeting NTD epitopes should be considered. NTD specific antibodies could be divided into two types: highly effective antivirus and low efficacy but polysaccharide-dependent neutralizing activity (dominant epitopes are RBD neutralizing epitopes, subdominant epitopes are neutralizing epitopes other than RBD) (81).

### Mutations in Other Regions

D614G mutation is an important mutation in SARS-CoV-2 and has become the dominant mutation site in all circulating SARS-CoV-2 variants (82, 83). Compared with D614, G614 could increase the viral load in the upper respiratory tract of patients but not in the lungs and may be conducive to the virus spread (84). Interestingly, G614 seemed to be more sensitive to neutralization by increasing the percentage of 1-RBD “up” conformation (85). In addition, neutralizing antibody titration (NT50) assay confirmed that no significant difference in neutralizing antibody titer between serum from the hamster infected with D614 strain and G614 strain (86). However, D614G mutation could not decrease the neutralization potency of most antibodies, indicating that this mutation is not the main immune escape site (29).

P681H/R mutations appeared in both B.1.1.7 and B.1.617 variants, and were proximal to the furin cleavage site (33), which could accelerate virus spread by increasing the membrane fusion rate (53). At present, the effect of P681H/R mutation on the affinity between virus and ACE2, and the neutralization potency of antibody are not clear. It is still necessary to carry out related tests and closely monitor the newly emerged mutations.

Residue 769 is located on the exposed S2 loop. In an immunocompromised SARS-CoV-2 infected person who was treated with convalescent serum, the variant harboring Δ69-70 and D769H was detected, and D769H was thought to be associated with immune escape (53).

## Impacts of Variants on Vaccines

As no effective drugs are available for SARS-CoV-2, vaccination becomes an important strategy in preventing and controlling the epidemic. The purpose of vaccination is to induce an immune response similar to natural infection and to produce associated immune cells and antibodies, and the antibody response caused by the vaccine is stronger than that caused by natural infection (36). Neutralization assay confirmed that the neutralization effect of three RBD mutations N439K, Y453F, and N501Y on convalescent serum was greater than that of vaccinated serum, which indicated that mRNA vaccine was more resistant to single RBD mutation than natural infection (9).

Since the outbreak of the SARS-CoV-2 epidemic, the world has accelerated the development process of vaccines (87). At present, there are seven kinds of potential SARS-CoV-2 vaccines: inactivated vaccines, live attenuated vaccines, DNA vaccines, mRNA vaccines, viral vectored vaccines, protein subunit vaccines, and virus-like particle vaccines. As of 26 September 2021, a total of 6,078,264,761 doses of vaccine [https://coronavirus.jhu.edu/map.html] have been administered worldwide. However, the emerging SARS-CoV-2 variants aroused great concern as a variety of vaccine sera showed weakened neutralization effect against variants (**Table 2**).

**Table 2:** The efficacy of approval vaccines against several prevalent variants.

Resource	Variant	Vaccine/Convalescent plasma	Sample	Conclusion
Wang et al. (24)	B.1.1.7	BBBIP-CorV	Vaccinated plasma	The neutralization titers cannot be detected in 6 of 25 BBBIP-CorV serum samples and 4 of 34 CoronaVac serum samples, respectively.
	B.1.351	(Sinopharm)		
		CoronaVac		
		(Sinovac)		
		BBBIP-CorV		The GMTs of neutralization against B.1.1.7 and B.1.351 were similar to WT, while 20 serum samples showed complete or partial loss of neutralization against B.1.351 variant.
		(Sinopharm)		
		CoronaVac		The GMTs of neutralization against B.1.1.7 and B.1.351 were significantly decreased than WT. Notably, most of the serum samples showed complete or partial loss of neutralization against B.1.351.
		(Sinovac)		
		Convalescent plasma	Convalescent plasma	The neutralization titers cannot be detected in 4 of 34 convalescent serum samples.
				The GMTs of neutralization against B.1.1.7 was similar to WT, the neutralization against B.1.351 was less effective. And 9 of 30 convalescent serum samples completely lost neutralization activity against B.1.351.
Supasa P et al. (22)	B.1.1.7	AZD1222	Vaccinated plasma	AZD1222 vaccinated sera showed a 2.5-fold reduction in neutralization activity against B.1.1.7 variant.
		(AstraZeneca)		
		BNT162b2		BNT162b2 vaccinated sera showed a 3.1-fold reduction in neutralization activity against B.1.1.7 variant.
		(Pfizer)		
		Convalescent plasma	Convalescent plasma	The neutralization activity of convalescent plasma against B.1.1.7 strain was 2.9-fold lower than those for the Victoria strain.
Madhi SA et al. (12)	B.1.351	AZD1222	Vaccinated plasma	The efficacy for the vaccine recipients is about 21%, which indicated that AZD1222 can’t provide a potent neutralization activity against B.1.351.
		(AstraZeneca)		
Wang P et al. (11)	B.1.1.7	mRNA-1273	Vaccinated plasma	Both of the vaccines showed essentially unchanged neutralization activity against B.1.1.7.
	B.1.351	(Moderna)		
		BNT162b2		
		(Pfizer)		
		Convalescent plasma	Convalescent plasma	Both of the vaccines showed significantly lower neutralization activity against B.1.351 (12.4-fold for the Moderna vaccine; 10.3-fold for the Pfizer vaccine).
				Most (16 out of 20) plasma samples lost more than 2.5-fold neutralizing activity against B.1.351, while maintaining the activity against B.1.1.7.
Liu et al. (88)	B.1.1.7	BNT162b2	Vaccinated plasma	The neutralization activity against B.1.1.7 was equal or higher than wildtype (USA-WA1/2020).
	B.1.351	(Pfizer)		
	P.1			
				The neutralization activity against B.1.351 and P.1 was reduced. The neutralization of B.1.351 was robust but lower.
Li et al. (29)	B.1.1.7	Convalescent plasma	Convalescent plasma	The plasma obtained from the 501Y.V2 infected patients showed a moderate efficacy against the first-wave virus while the plasma obtained from the first-wave virus infected patient could not neutralize 501Y.V2.
	B.1.351			
				The efficacy of the plasma elicited by the first-wave against the first-wave virus is better than that of the plasma elicited by 501Y.V2 infected patients. The similar circumstance happened in the 501Y.V2 case.
Li et al. (29)	B.1.351	Convalescent plasma	Convalescent plasma	Mutations at a single site would not lead to significant alteration of the neutralization activity.
				E484K and N501Y mutations resulted in a significant decrease in neutralization, which implied these two sites were situated in immunodominant epitopes.
				Notably, the presence of K417N apparently increases susceptibility to neutralization by polyclonal antibodies.
Yadav PD et al. (89)	B.1.1.7	BBV152	Vaccinated plasma	The neutralization activity against B.1.617 was weaker by approximately 2-fold than the D614G mutant. The neutralization against B.1.1.7 was significantly higher than B.1.617.
	B.1.617			
		Convalescent plasma	Convalescent plasma	The neutralizing capacity of convalescent plasma and vaccinated plasma against B.1.617 were similar.
McCallum M et al. (36)	B.1.427/B.1.429	mRNA-1273	Vaccinated plasma	Both of the vaccines showed lower neutralization activity against B.1.427/B.1.429 (2.8-fold for the Moderna vaccine; 4-fold for the Pfizer vaccine) than the D614G variant.
		(Moderna)		
		BNT162b2		
		(Pfizer)		
Shen X et al. (9)	B.1.1.7	mRNA-1273	Vaccinated plasma	Compared with D614G mutation, neutralization titer of ID50 against B.1.1.7 decreased 2.0-fold, the neutralization titer of ID80 decreased by 1.7-fold.
		(Moderna)		
		NVX-CoV2373, (Novavax)		Compared with D614G mutation, B.1.1.7 ID50 neutralization titer decreased by 2.1-fold, and the neutralization titer of ID80 decreased by 1.8-fold.
Collier DA et al. (8)	B.1.1.7/B.1.1.7 with E484K	BNT162b2	Vaccinated plasma	The mean fold change for the E484K-containing B.1.1.7 spike variant was 6.7 compared with 1.9 for the B.1.1.7 variant, relative to the WT spike protein.
		(Pfizer)		
A DC et al. (90)	B.1.351	The Novavax COVID-19 vaccine	Vaccinated plasma	In the South Africa trial of over 4,400 people, the vaccine was 60% effective in people that were HIV negative, compared to 89.3% effective at preventing COVID-19 in participants in its Phase 3 clinical trial in the UK.
		the Johnson and Johnson, JNJ vaccine		In a Phase 3 trial including 44,000 people, a single dose of the Johnson and Johnson, JNJ vaccine showed an overall protective efficacy of 66%.
Dejnirattisai W et al. (5)	P.1, B.1.1.7, B.1.351	Convalescent plasma	Convalescent plasma	Compared with Victoria, P.1 geometric mean neutralization titers were reduced by 3.1-fold, B.1.1.7 were reduced by 2.9-fold and B.1.351 were reduced by 13.3-fold.
			B.1.1.7 convalescent plasma	Compared with Victoria, P.1 geometric mean neutralization titers were reduced by 1.8-fold, B.1.1.7 were reduced by 1.1-fold and B.1.351 were reduced by 4.4-fold.
		BNT162b2	Vaccinated plasma	Geometric mean neutralization titers against P.1 were reduced by 2.6-fold relative to the Victoria virus, B.1.1.7 were reduced by 3.3-fold and B.1.351 were reduced by 7.6-fold.
		(Pfizer)		
		ChAdOx1 nCoV-19(Oxford-AstraZeneca)	Vaccinated plasma	Geometric mean neutralization titers against P.1 were reduced by 2.9-fold, B.1.1.7 were reduced by 2.3-fold and B.1.351 were reduced by 9-fold.
Zhou D et al. (59)	B.1.351	Convalescent plasma	Convalescent plasma	Neutralization titers against B.1.351 were, on average, 13.3-fold reduced compared with Victoria.
		B.1.1.7 convalescent plasma	B.1.1.7 convalescent plasma	Overall, there was a 3.1-fold reduction in titers between Victoria and B.1.351 in sera from patients infected with B.1.1.7.
		BNT162b2	Vaccinated plasma	Geometric mean titers for B.1.351 were 7.6-fold lower than for Victoria.
		(Pfizer)		
		AZD1222	Vaccinated plasma	Geometric mean B.1.351 titers were 9-fold lower than for Victoria.
		(Oxford-AstraZeneca)		
Baoying Huang et al. (91)	B.1.351	BBIBP-CorV	Vaccinated plasma	ZF2001 or BBIBP-CorV remained protective against B.1.351 with the potential
		RBD ZF2001		
				1.6-fold reduction of neutralizing GMTs.
Wu K et al. (92)	B.1.1.7	mRNA-1273	Vaccinated plasma	B.1.1.7 variant had no significant neutralization effect on serum from vaccinated participants, the titers of neutralizing antibodies against the P.1, B.1.427/B.1.429, B.1.351 variant reduced by a factor of between 2.3 and 6.4.
	B.1.351	(Moderna)		
	P.1			
	B.1.427/4			
	29			
Garcia-Beltran WF et al. (17)	B.1.1.7	BNT162b2	Vaccinated plasma	Neutralization titers against B.1.1.7, B.1.351 and P.1 were reduced by 2.1-fold, 34.5~42.4-fold and 6.7-fold.
	B.1.351	(Pfizer)		
	P.1			
		mRNA-1273		The neutralization titers against B.1.1.7, B.1.351 and P.1were reduced by 2.3-fold 19.2~27.7-fold and 4.5-fold.
		(Moderna)		
Wang P et al. (11)	B.1.1.7	mRNA-1273	Vaccinated plasma	Compared with D614 mutation, the neutralization activity of mRNA-1273 against B.1.1.7 mutant decreased by 1.8-fold and that against B.1.351 decreased by 8.6-fold. Similarly, the neutralization activity of BNT162B2 against B.1.1.7 mutant decreased by 2-fold, and that against B.1.351 decreased by 6.5-fold.
	B.1.351	BNT162b2		

### Inactivated Vaccines

Inactivated vaccine is the most classical vaccine form, easy to prepare and can cause effective immune response (93). In clinical phase I and phase II trials, BBIBP-CorV vaccine produced extensively high titer of neutralization antibodies (94). At present, three inactivated vaccines independently developed by China have been put into use. However, it may be difficult to protect the variants since the seed strain of inactivated vaccine is from the Wuhan virus. Wang et al. evaluated the neutralization effect of two inactivated vaccines BBIBP-CorV and CoronaVac against B.1.1.7 and B.1.351 by SARS-CoV-2 pseudoviruses. The results showed that BBIBP-CorV could still retain a neutralization effect against B.1.1.7, but 20 of 25 BBIBP-CorV serum samples were ineffective or partially lost activity against B.1.351. In addition, the neutralization effects of CoronaVac against B.1.1.7 and B.1.351 variants were impaired than that of the WT strain. In addition, no neutralization antibody was detected in 6 of 25 BBIBP-CorV serum samples and 4 of 34 CoronaVac serum samples (24). This result indicated that antibody response heterogeneity exists between individuals after vaccination. Therefore, prevention and control measures such as wearing masks and keeping social distance should still be maintained in risky areas. Yadav PD et al. investigated the inactivated vaccine BBV152 and found that compared with WT strain, the neutralization activity of BBV152 vaccine against B.1.617 decreased by about 2-fold, but it was able to potently neutralize B.1.1.7 (89). All of the above findings indicated that the mutation should be monitored continuously, and new seed strains of SARS-CoV-2 could be considered to update the existing inactivated vaccine (21, 95).

### Viral Vectored Vaccines

The vectored vaccines are made by using viruses or bacteria as vectors and inserting genes encoding effective immunogens of pathogens into the vector. Usually, most of the marketed vector vaccines targeting SARS-CoV-2 employ adenoviruses vectors (e.g., vaccines from ConSino and AstraZeneca), which can induce both innate immunity and adaptive immunity (96).

The neutralizing activity of the AZD1222 vaccine against B.1.1.7 and B.1.351 strains was evaluated, and the results showed that widespread immune escape was not observed in B.1.1.7, while greater resistance to B.1.351 was observed in both the pseudovirus and the live-virus neutralization assays (12, 22). Therefore, it is still necessary to update the immunogen according to the virus mutation. Real-world research has confirmed that the protective effectiveness of AstraZeneca vaccine against B.1.617.2 strain is 59.8%, while that of B.1.1.7 strain is 66.1%, suggesting that B.1.617.2 has stronger immune escape ability than B.1.1.7 (97). The ChAdOx1 nCoV-19 vaccine was still effective against the B.1.1.7 variant in clinic, but it had a poor protective effect on mild-to-moderate diseases caused by the B.1.351 variant (12, 98, 99).

### Protein Subunit Vaccines

Protein subunit vaccines use specific protein regions of pathogens to exert immunogenicity. Such vaccines only hold the necessary antigens related to infection and have fewer side effects on the body (100). At present, NVX-CoV2372 vaccines comprise S protein in full-length for vaccine antigen component in clinical use, ZF2001 vaccines apply the dimer form of RBD as vaccine antigen component, while Pfizer and Moderna utilize the trimeric RBD (101, 102).

Huang et al. evaluated the neutralization effects of BBIBP-CorV and ZF2001 on B.1.351. The results showed that although the antibody titer of these vaccinated sera against B.1.351 decreased slightly compared with WT, both of them could effectively neutralize B.1.351 (91). NVX-CoV 2373 (Novavax) protein vaccines provided 95.6% protection efficacy against WT virus, 85.6% efficacy against the B.1.1.7 variant and 60.0% efficacy against the B.1.351 variant (99). Similarly, single-dose vaccination of JNJ-78436735 (Johnson/Janssen) remained 72% protective effect on moderate-to-severe COVID-19 patients in the United States, but in South Africa, where B.1.351 strain had been widely prevalent, it only had a 57% protective effect on moderate-to-severe SARS-CoV-2 infection (103). These results suggest that the protein subunit vaccines have a better neutralization effect on WT and B.1.1.7 strains, and the effect on highly mutated variants including B.1.351 should be monitored continuously.

### Nucleic Acid Vaccines

Nucleic acid vaccines can be divided into RNA vaccines and DNA vaccines. At present, most nucleic acid vaccines for SARS-CoV-2 are mRNA vaccines. These kind of vaccines have been confirmed to be durable, effective and safe in animal experiments, and could simultaneously induce both T and B cell immune responses in the body (104). Supasa P et al. evaluated the neutralizing response of serum from BNT162b2 vaccines against the B.1.1.7 variant, the neutralization activity decreased by 3.3-fold compared to the WT strain, and no immune escape was observed (22). Real-world studies in Qatar showed that the protective efficacy of BNT162b2 vaccines against B.1.1.7 variant was estimated to be 87.0%, and the efficacy against B.1.351 variant was estimated to be 72.1%. Fortunately, its protection against severe disease was still above 90% (105). Similar to the vector vaccines, the neutralization activity of mRNA vaccines against B.1.351 variant was significantly reduced (11, 88, 106). In a study conducted in Israel, BNT162b2 vaccine could still effectively neutralize most of the variants (e.g., B.1.427/B.1.429, B.1.526) (88, 107). Accordingly, the neutralization potency assay using pseudoviruses found that compared with WT (D614), the average neutralization potency of BNT162B2 vaccinated sera decreased by 2.8-fold for B.1.427/B.1.429, 3.2-fold for B.1.351, 1.2-fold for B.1.1.7 and 1.7-fold for P.1, respectively (36). In addition, mixed use of mRNA vaccines seemed to be an effective method. The novel mRNA vaccine mRNA-1273.211, which is a mixture of mRNA-1273 and vaccine encoding B.1.351 S protein, has stronger neutralization efficacy against B.1.351 and P.1 than the monovalent vaccine in animal experiments, and is still valid for B.1.427/429 (108). Compared with mRNA vaccines, DNA vaccines have higher stability and can be stored for a long time (109), as of September 26, 2021, 11 DNA vaccines have been approved for clinical trials [https://www.who.int/emergencies/diseases/novel-coronavirus-2019/covid-19-vaccines]. Momin T et al. reported the results of phase I clinical trial of ZyCoV-D vaccine, a DNA candidate vaccine composed of plasmid DNA carrying RBD gene of Spike and signal peptide gene. The study revealed that this vaccine had good safety, but the serum titer was less than convalescent serum (110). In addition, another DNA candidate vaccine INO-4800, based on full-length of spike protein, showed higher efficiency by the inoculation method of electroporation, and induced neutralizing antibodies in all vaccinated cases (111). Compared with mRNA vaccines, the titers of neutralizing antibody induced by DNA vaccines are still low (112, 113). Thus, it is necessary to improve the efficiency of DNA vaccines and optimize the inoculation method.

### Virus-Like Particle Vaccines

The prevalence of variants triggers significant challenges in vaccines design. Compared with conventional subunit vaccines with non-granular antigens, polyvalent nanoparticles usually significantly enhance neutralizing antibody responses, and polyvalent RBD nanoparticles using two-component protein nanoparticles I53-50 have strong antigenic effects, eliciting strong antibody responses against multiple epitopes (114). Compared with the vaccine based on soluble S protein, the new nanoparticle vaccine produced 10-fold more neutralizing antibodies in mice (114). Another study also developed a nano-vaccine supplemented with 3M-052 adjuvant to induce protective immunity against SARS-CoV-2 and a variety of β-coronaviruses (115).

## Impacts of Variants on Antibodies and Monoclonal Antibodies

**Figure 6:**
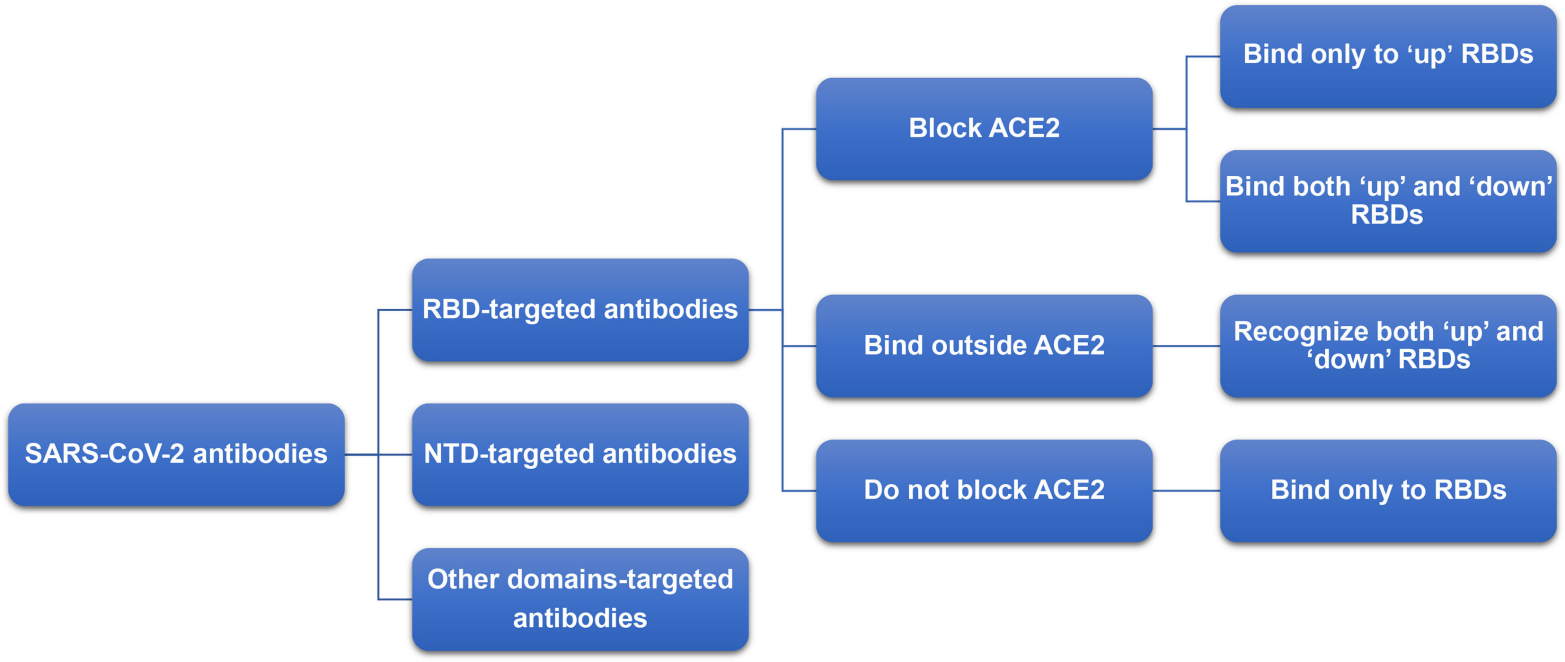
Classification of neutralizing antibody targeting RBD. The neutralizing antibodies targeting SARS-CoV-2 RBD can be classified into four categories: (1) block ACE2 and bind only to ‘up’ RBDs; (2) block ACE2 and can bind to both ‘up’ and ‘down’ RBDs and can contact adjacent RBDs; (3) bind outside the ACE2 sites and recognize both ‘up’ and ‘down’ RBDs; (4) bind to the external residue sites of ACE2 and bind only to ‘up’ RBDs.

Antibodies are important tools for the treatment of infectious diseases (116). Most of the antibodies used in the treatment of COVID-19 target RBD or NTD, as these two regions have higher immunogenicity (117). The neutralizing antibodies targeting SARS-CoV-2 RBD can be classified into four categories (**Figure 6**) (54). Among these categories, the immune superiority of RBM epitopes directly contacting with ACE2 are more obvious than other epitopes (56). The mechanism of monoclonal antibodies targeting NTD is still not clear (54, 118).

Both antibody therapy and convalescent serum therapy can lead to higher immune pressure (118). Constantly subjected to immune pressure caused by the same class of neutralizing epitopes leads to the emergence of novel mutations in these neutralizing epitopes, providing the possibility of antibody response escape (**Table 3**). However, the simultaneous use of two antibodies targeting different epitopes will help to alleviate this situation, because viruses with simultaneous double-site mutations are not easy to survive under such conditions (73, 119). In addition, due to the key role of S2 subunit in membrane fusion (120), and the high conservation of S2 sequence, antibodies or vaccines targeting fusion peptide (FP) can be designed to reduce mutations caused by immune pressure and enhance the persistence and effectiveness of antibodies and vaccines. Antibodies targeting FP can play a role by preventing protease-mediated cleavage of S2 site. In view of the fact that RBD has a high mutation entropy, which increases the possibility of vaccine-induced immune escape, these conservative targets in S2 may be considered for future vaccine design (121, 122).

**Table 3:** The efficacy of antibodies against several prevalent variants.

Resource	Site	Antibody	Type	Conclusion
McCarthy KR et al. (33)	NTD	4A8	mAbs	The deletions of 69-70 and 144-145 positions completely abolished binding of 4A8.
Shen X et al. (9)	RBD	CoV2-15 and B38	mAbs	The B.1.1.7 variant showed greatest resistance to mAbs B38, COVA2-15, and S309 (>10-fold difference in either IC50 or IC80 concentration compared to D614G), the resistance to COVA2-15 was largely due to N501Y.
McCallum M et al. (36)	L452R	bamlanivimab (LY-CoV555)	RBD targeting mAbs	1.The single L452R mutation present in the SARS-CoV-2 B.1.427/B.1.429 S RBD leads to a reduction or abrogation of the neutralizing activity of 10 out of 34 RBD-specific mAbs evaluated, including regdanvimab (CT-P59), etesevimab (LY-CoV016) and bamlanivimab (LY-CoV555). Bamlanivimab (LY-CoV555) entirely lost its neutralizing activity due to the central location of L452R in the epitopes recognized by these mAbs. Regdanvimab (CT-P59), and to a smaller extent etesevimab, showed a reduction in neutralization potency. Neutralization mediated by the casirivimab/imdevimab mAb cocktail (REGN10933 and REGN10987) and by VIR-7831 mAb is unaffected by the L452R mutation.
		Regdanvimab (CT-P59)		
		etesevimab		
		casirivimab/imdevimab		
		VIR-7831		
		S2D8 S2D19 S2D32 S2D97 S2E12 S2H7 S2H14 S2H19 S2H58 S2H71 S2M11 S2N28 S2X128 S2X192 S2X259 S2X615 S2H70 S2N12 S2N22 S2X608 S2X609 S2X30 S2X305 S2D106 S2X619 S2X58 S2H94 S2H97		
	S13I/W152C	4A8 S2L26 S2L50 S2M28 S2X28 S2X303 S2X158 S2X107 S2X333 S2X124	NTD targeting mAbs	The neutralizing activity of all NTD-specific neutralizing mAbs tested was abolished as a result of the presence of the S13I and W152C mutations.
Graham C et al. (81)	ΔY144	S2M28、S2X28、S2X333and 4A8	NTD targeting mAbs	The ΔY144 deletion has been shown to abrogate binding to other NTD mAbs includingS2M28, S2X28, S2X333, and 4A8.
	B.1.351	4A8	NTD targeting mAbs	Deletion of NTD residues 242–244 from the B.1.351 variant (501Y.V2 prevalent in South Africa) has been shown to reduce binding by NTD-specific mAbs 4A8 and 4-8.
Supasa P et al. (22)	B.1.1.7	IGHV3-53	RBD targeting mAbs	For some antibodies (40, 88, 222, 316, 384, 398), the neutralization activity against B.1.1.7 were minimally affected (< 2-fold difference).
		IGHC1-58		
		IGHV3-66		
		REGN-CoV		
				For others, there was a fall in the neutralization titres for B.1.1.7. Notably, the mAb 269, completely lost the neutralization and the neutralization of mAb 278 showed a maximum of only 78%.
				For the EUA (Emergency Use Authorization) antibodies (REGN-CoV), the neutralization of REGN10987 was unaffected by B.1.1.7 while REGN10933 showed a slight reduction but still retained potent activity.
Collier DA et al. (8)	B.1.1.7	S2X192	RBD targeting mAbs	Neutralization of 5 monoclonal antibodies that target the RBM showed more than 100-fold decrease against B.1.1.7.
		S2H14		
		S2H19		
		S2D8		
		S2X107	NTD targeting mAbs	The B.1.1.7 variant fully escaped neutralization by 8 monoclonal antibodies that target the NTD.
		S2X28		
		S2X333		
		S2X158		
		4A8		
		S2X124		
		S2L26		
		S2X303		
Wang P et al. (11)	B.1.1.7	2-15	RBD targeting mAbs	Only the activities of 910-30 and S309 against B.1.1.7 are substantially impaired, other RBD mAbs still maintain the efficacy.
	B.1.351	REGN10933		
		C121		
		LY-CoV-555		
		2-36		
		COVA1-16		The activities of 910-30, 2-15, LY-CoV555, C121 and REGN10933 (4 of which target RBM) against B.1.351 are completely or markedly abolished.
		910-30		
		2-7		
		REGN10987		
		1-57		
		C135		The activities of 2-36, COVA-1, 2-7, REGN10987, C135 and S309 (outer or inner side) still retained.
		S-309		
				The complete loss of activity of 2-15, LY-CoV555 and C121 against B.1.351 is mediated by the E484K substitution.
				The complete loss of activity of 910-30 is mediated by the K417N substitution; and the marked reduction in activity of REGN10933 is mediated by K417N and E484K.
		5-24	NTD targeting	Both B.1.1.7 and B.1.351 are markedly resistant to neutralization by antibodies 5-24, 4-8 and 4A8.
		4-8		
		4A8	mAbs	
		2-17		
		4-19		The resistance of B.1.1.7 to most NTD-directed monoclonal antibodies is largely conferred by ΔY144, whereas the resistance of B.1.351 is largely conferred by Δ242–Δ244 and/or R246I.
		5-7		
		1-20	RBD	The activity of CB6 is rendered inactive against B.1.351.
		4-20	targeting	
		2-4	mAbs	The efficacy of Brii-196 and COV2-2130 are essentially unaffected by B.1.351.
		2-43		
		2-30		
		2-38		The activities of Brii-198 and COV2-2196 against B.1.351 are diminished by 14.6-fold and 6.3-fold, respectively.
		CB6		
		COV2-2196		
		COV2-21307		
		Brii-196		The activity of CB6 is rendered inactive against B.1.351 because of the K417N substitution.
		Brii-198		
		REGN10985		
McCallum M et al. (32)	B.1.1.7	S2L28	NTD	S2L28, S2M28, S2X28, and S2X333 efficiently blocked cell-cell membrane fusion of Vero E6 cells transiently transfected with full-length wild-type SARS-CoV-2 Spike protein.
		S2M28	targeting	
		S2X28	mAbs	
		S2X333		
				Currently circulating variants will partially or completely escape neutralization mediated by mAbs targeting the antigenic supersite (site i), including the B.1.1.7, B.1.351, and P.1 lineages.
Li Q et al. (29)	B.1.351	10D12	RBD	The neutralization activity of most mAbs (12 of 17) are affected.
		11D12	targeting	
		247	mAbs	
		157		No alteration of neutralization sensitivity was observed for 5 of the 17 monoclonal antibodies: 2F7, P2C-1F11, H014, 4E5, and 7B8.
		2H10		
		1F9		
		261-262		
		9G11		
		P2B-2F6		Mutations at a single site did not lead to significant alteration of the neutralization activity of polyclonal antibodies.
		LKLH		
		H00S022		
		10F9		
				Only the simultaneous presence of the E484K and N501Y mutations resulted in a significant decrease in neutralization.
Dejnirattisai W et al. (5)	P.1	150, 158, 175, 222, 269, 40, 398, 55, 58, 88, 132, 159, 165, 170, 253, 278, 281, 316, 318, 384	RBD targeting	Compared to Victoria, mAbs neutralization was significantly impacted by P.1, with 12/20 showing > 10-fold reduction in FRNT50 titer and a number showing complete knockout of activity. The results with P.1 showed a greater impact compared to B.1.1.7 but similar to those with B.1.351. mAb 222 neutralizes all three variants.
	B.1.351			
	B.1.1.7		mAbs	
		S309, AZD8895, AZD1061, AZ7442, REGN10987, REGN10933, LY-CoV555, LY-CoV16, ADG10, ADG20, and ADG30	RBD targeting	S309 was largely unaffected. AZD8895 showed modest reduction in neutralization of P.1. REGN10933 was escaped from P.1 LY-CoV16 and LY-CoV555 showed almost complete loss of neutralization of P.1 and B.1.351. LY-CoV16 also showed marked reduction in neutralization of B.1.1.7. Three Adagio antibodies neutralized all variants.
			mAbs	
		mAb159	NTD targeting	The neutralization titer of mAb159 was 133-fold reduced on P.1 compared to Victoria.
			mAbs	
Zhou D et al. (59)	B.1.351	40, 55, 58, 88, 132, 150, 158, 159, 165, 170, 175, 222, 253, 269, 278, 281, 316, 318, 384, 398	RBD targeting mAbs	The effects of B.1.351 on mAb neutralization were severe, 14 of 20 antibodies had >10-fold fall in neutralization titers, with most of these showing a complete knockout of activity.
		REGN10933, REGN10987, AZD106 and AZD8895	RBD targeting	The neutralization of REGN10987 was unaffected by B.1.351, while REGN10933 was severely impaired (773-fold). Neutralization by the AZ pair of antibodies was little affected on B.1.351 compared with Victoria.
			mAbs	
		mAb 159	NTD targeting mAbs	The mAb 159 showed a complete knockout of activity against B.1.351.

At present, most of the monoclonal antibodies (mAbs) used in clinical (e.g., REGN10933, LY-CoV-555) still maintain high neutralization efficacy against B.1.1.7 (11), but most of the mAbs showed a remarkable decrease against B.1.351 (11, 29). mAbs therapy can produce selective pressure, which would increase the possibility of virus escaping from targeted antigen mutations. The combination of two or more mAbs that target non-overlapping epitopes could reduce the escape possibility (123). Moreover, due to the key roles of L452R, LY-CoV-555 completely lost the neutralization activity to B.1.427/B.1.429 (36). Other studies showed that the cocktail therapy was less affected by B.1.1.7 (22). Therefore, cocktail therapy may become an effective method for the treatment of SARS-CoV-2 variants infection (73, 119). The non-overlapping epitopes and lack of binding competition are the critical factors in mAbs cocktail selection (73). According to this principle, some studies have designed the combination of CoV2-06 and CoV2-14 as cocktail antibodies. The targeted epitopes of CoV2-06 and CoV2-14 were mutated at the same time, which was not conducive to virus survival. Therefore, the epitopes of CoV2-06 and CoV2-14 can be considered in future vaccine design so as to reduce the immune escape of variants (73). Similarly, COVID-19 IgGs can be obtained from the isolated convalescent serum. These IgGs can target different epitopes of S protein to play effective neutralizing roles (124).

In addition, some mAbs have cross neutralization activity such as COVA1-16, which can maintain neutralization activity against a variety of variants (e.g., B.1.1.7, B.1.351) by binding to highly conserved epitopes of S protein (125). The infection of B.1.1.7 or B.1.351 can also cause low levels of cross neutralization antibodies (30, 36).

Notably, Sinopharm announced that they have isolated a potent monoclonal antibody 2B11 with higher neutralization activity from the convalescent sera. The IC50 of antibody 2B11 against Delta variant is 5 ng/mL, which is a promising alternative treatment for COVID-19 (126).

## Conclusion and Perspectives

Continuous surveillance of viral genome sequence data and the effectiveness of vaccines would help to better understand the drivers of SARS-CoV-2 transmission and evolution, providing a basis for vaccine development and update (123). The same point mutation (e.g., D614G, N501Y) sequenced in different virus strains began to spread globally, suggesting that these mutations have certain evolutionary advantages (118). Two important determinants of variant spread are occurrence frequency in individuals and transmission possibility. Immune escape mutations in a single host may be relatively rare, at least in early infections. However, potential host adaptive mutations can be observed even in the absence of vaccine or antibody therapy selection pressure. It is suggested that transmission-enhancing and/or immune-escape SARS-CoV-2 variants are unlikely to arise frequently, but could spread rapidly if they are successfully transmitted (65). We speculate that early mutations of the virus such as B.1.1.7 mainly enhance its transmission ability, while in the later stage of the epidemic, immune escape strains start to emerge with the increased immune pressure.

The number of people with acquired immunity against COVID-19 continues to increase after natural infection or vaccination, but due to unequal interventions and access to vaccines, the virus would subject to greater immune pressure and require repeated immunization rounds to deal with the continuous arising of virus variants (127). Postponement of the second dose could help more people get vaccination when the vaccine production dose is not enough, but the proposal to change the vaccine scheme to a single dose may accelerate the evolution of virus strains. The antibody titer produced by only one dose of vaccination would not be sufficient for virus infection prevention and virus clearance, which is likely to contribute to the production of vaccine-resistant strains (118).

The emergence of new variants emphasizes the need for continued vigilance. Since vaccine-induced herd immunity increases the probability of immune escape, it is difficult to determine which variants or sequences should be selected to update the vaccine sequence. B.1.351 is the variant of greatest concern, with the strong resistance to vaccine serum and antibodies. Therefore, it is believed that the development of vaccine constructs using B.1.351 is the top priority (5). As COVID-19 continues to spread, more virus variants with the ability to escape the neutralization of antibodies would appear. The protection of these variants can be ensured by the combination of two or more potent neutralizing antibodies against different epitopes (128). In addition, using antibody cocktails to resist virus mutation seems to be a sensible strategy. However, it must be recognized that the use of mAbs for long-term treatment or prevention, especially in chronic infected individuals who are immunocompromised, may lead to the emergence of neutralization resistance mutations. In order to avoid selective pressure and immune escape, it is suggested that antibody therapy might consist of a combination of antibodies targeting non-overlapping or highly conserved epitopes (129). Considering the important role of site 484 for antibody binding and neutralization, it is a good strategy to identify monoclonal antibodies from E484K infected individuals (59).

The global circulation of multiple SARS-CoV-2 variants undermines confidence in whether the current vaccines will provide long-term protection. The vaccines elicited antibody responses against RBD in a manner similar to natural infection (63), suggesting that vaccines could reduce the severity of disease caused by natural infection more or less. In addition, the response of T cells against spikes cannot be disturbed by mutations and could still prevent severe diseases caused by variants (59). Moreover, under the continuous emergence of SARS-CoV-2 mutations, the body’s immune system is also constantly improving its ability to deal with the evolution. The memory B cells did not decrease after 6.2 months of effective vaccination, but continued to evolve and were involved in preventing reinfection. These results strongly suggest that vaccinated individuals can respond quickly and effectively to the virus upon exposure (130).

New variants will continue to emerge (123), and intensive surveillance systems are needed to monitor the arising of new variants. Moreover, breakthrough infections among vaccinees urgently need to be elucidated. The second or even third generation vaccines targeting virus variants, as well as the more extensive development of immunogens targeting ACE2-RBD-independent surfaces, are worthy of further study (59).

## Author Contributions

HF, JF, and ML designed the research. FL, ML, ZP, LJ, LG, JF, and HF read and analyzed the papers. LT and JH participated in discussion. FL, ML, JF, and HF wrote and revised the manuscript. All authors contributed to the article and approved the submitted version.

## Funding

This manuscript was funded by grants from National Key Research and Development Program of China (grant No. 2020YFA0712102), Fundamental Research Funds for Central Universities (grant No. BUCTZY2022), and H&H Global Research and Technology Center (grant No. H2021028).

## Conflict of Interest

The authors declare that the research was conducted in the absence of any commercial or financial relationships that could be construed as a potential conflict of interest.

## Publisher’s Note

All claims expressed in this article are solely those of the authors and do not necessarily represent those of their affiliated organizations, or those of the publisher, the editors and the reviewers. Any product that may be evaluated in this article, or claim that may be made by its manufacturer, is not guaranteed or endorsed by the publisher.

## Supplementary Material

Supplementary Figure 1 Brief information of six former VOI variants. Six former VOC variants (B.1.427/429, P.2, P.3, B.1.525, B.1.526, and B.1.617.1) are marked in the arrow according to the date of designation, and their related brief information (e.g., the time and location of earliest documented samples, infectivity, main mutations, immune escape ability) are displayed in the corresponding location.

Supplementary Figure 2 Mutations of six former VOI variants. Schematic showing the locations of amino acid substitutions of six former VOI variants (B.1.427/429, P.2, P.3, B.1.525, B.1.526, and B.1.617.1) in spike protein. The RBD region is shown in modena, the NTD region is shown in shallow orange.
