# Supplementary figures and images for: Understanding the Secret of SARS-CoV-2 Variants of Concern/Interest and Immune Escape
Source: Front Immunol. 2021 Nov 5;12:744242. doi: 10.3389/fimmu.2021.744242 (PMC8602852; doi:10.3389/fimmu.2021.744242)

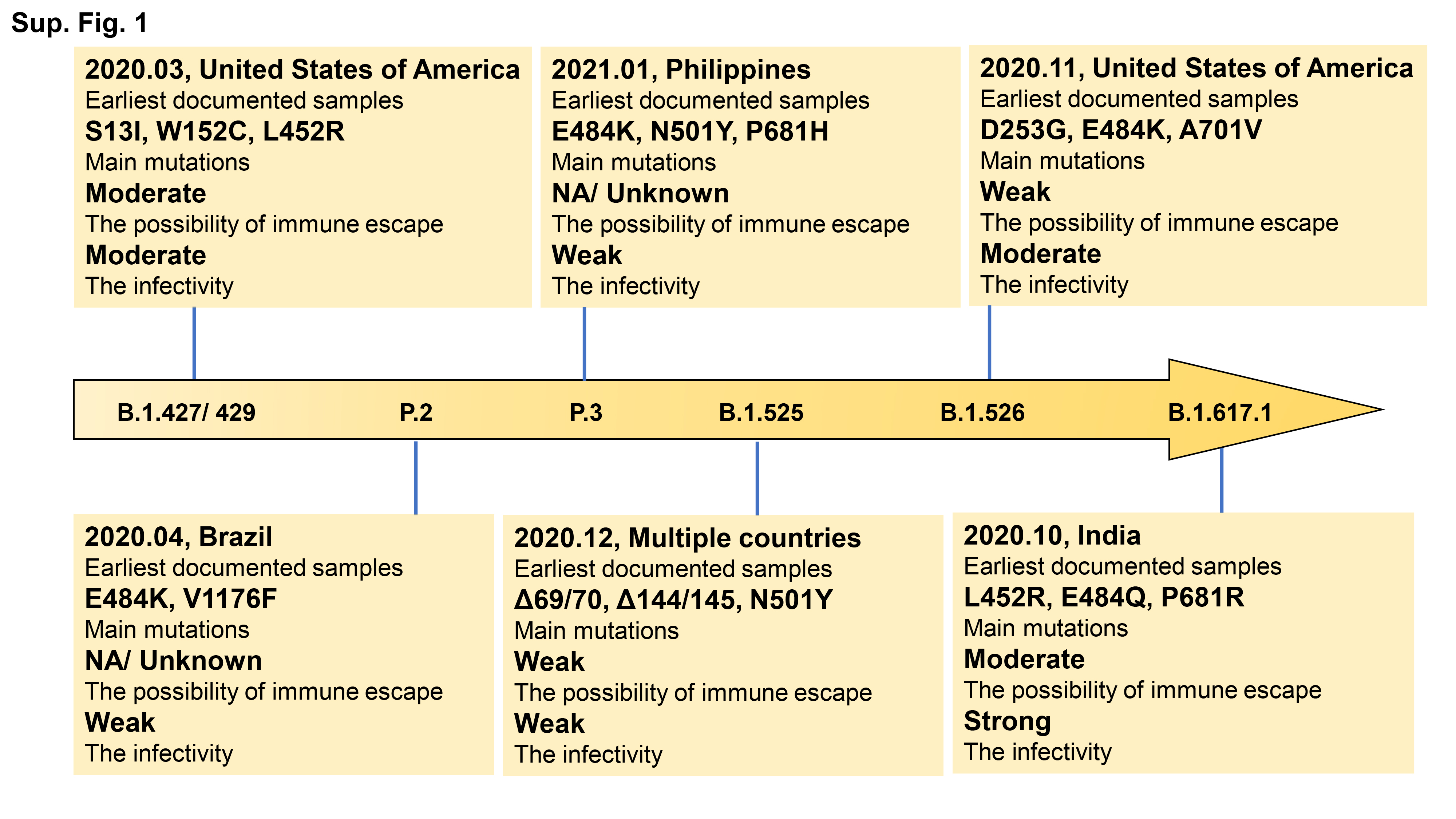

Supplement: Supplementary Figure 1 — Brief information of six former VOI variants. Six former VOC variants (B.1.427/429, P.2, P.3, B.1.525, B.1.526, and B.1.617.1) are marked in the arrow according to the date of designation, and their related brief information (e.g., the time and location of earliest documented samples, infectivity, main mutations, immune escape ability) are displayed in the corresponding location. [file Image_1.jpeg]

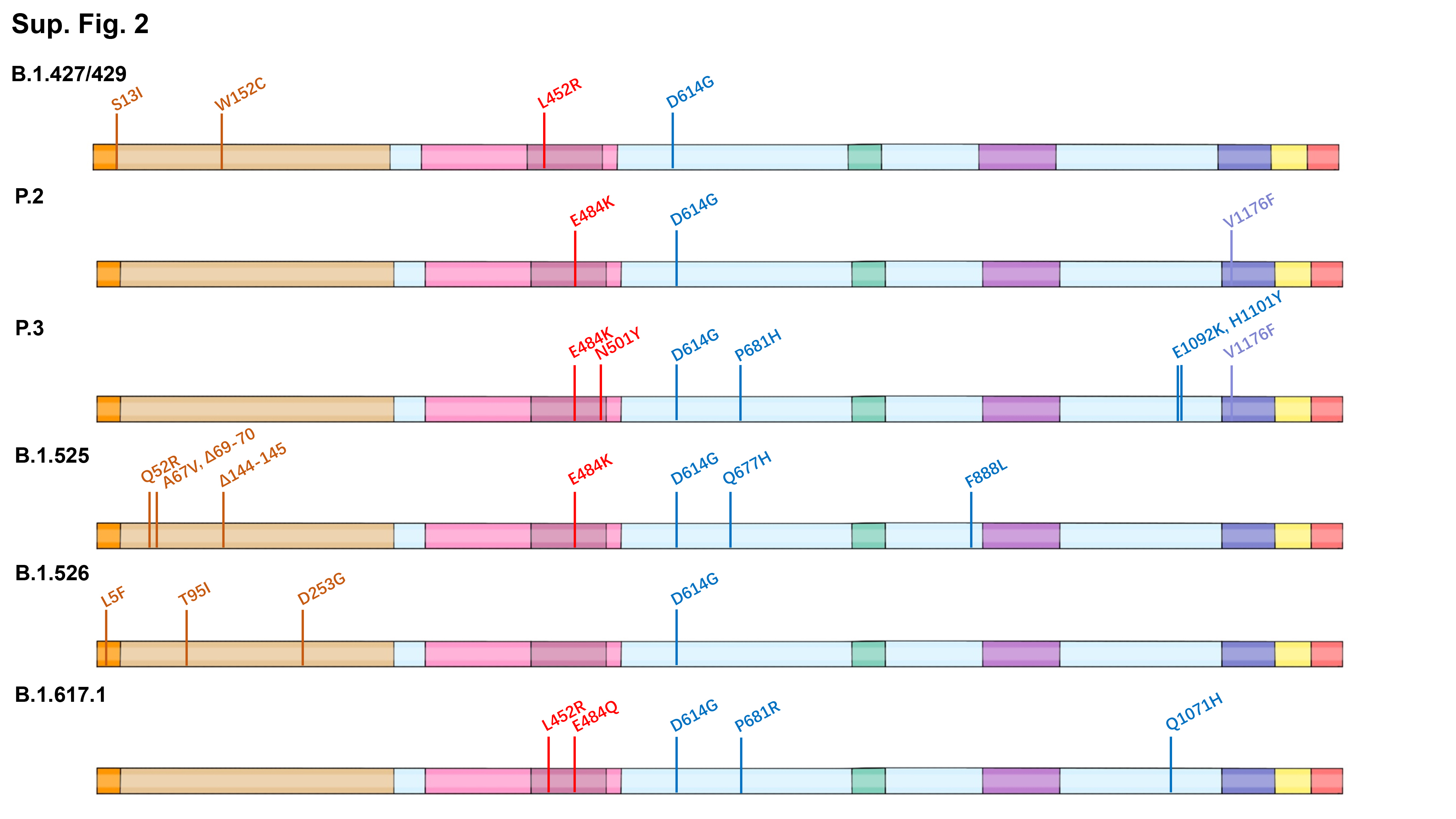

Supplement: Supplementary Figure 2 — Mutations of six former VOI variants. Schematic showing the locations of amino acid substitutions of six former VOI variants (B.1.427/429, P.2, P.3, B.1.525, B.1.526, and B.1.617.1) in spike protein. The RBD region is shown in modena, the NTD region is shown in shallow orange. [file Image_2.jpeg]
